# Osmotic stress‐responsive promoter upstream transcripts (PROMPTs) act as carriers of MYB transcription factors to induce the expression of target genes in *Populus simonii*

**DOI:** 10.1111/pbi.12955

**Published:** 2018-06-28

**Authors:** Yuepeng Song, Anran Xuan, Chenhao Bu, Dong Ci, Min Tian, Deqiang Zhang

**Affiliations:** ^1^ Beijing Advanced Innovation Center for Tree Breeding by Molecular Design Beijing Forestry University Beijing China; ^2^ National Engineering Laboratory for Tree Breeding College of Biological Sciences and Technology Beijing Forestry University Beijing China; ^3^ Key Laboratory of Genetics and Breeding in Forest Trees and Ornamental Plants College of Biological Sciences and Technology Beijing Forestry University Beijing China

**Keywords:** PROMPTs, lncRNAs, osmotic stress‐responsive, *Populus simonii*

## Abstract

Complex RNA transcription and processing produces a diverse range catalog of long noncoding RNAs (lncRNAs), important biological regulators that have been implicated in osmotic stress responses in plants. Promoter upstream transcript (PROMPT) lncRNAs share some regulatory elements with the promoters of their neighbouring protein‐coding genes. However, their function remains unknown. Here, using strand‐specific RNA sequencing, we identified 209 differentially regulated osmotic‐responsive PROMPTs in poplar (*Populus simonii*). PROMPTs are transcribed bidirectionally and are more stable than other lncRNAs. Co‐expression analysis of PROMPTs and protein‐coding genes divided the regulatory network into five independent subnetworks including 27 network modules. Significantly enriched PROMPTs in the network were selected to validate their regulatory roles. We used delaminated layered double hydroxide lactate nanosheets (LDH‐lactate‐NS) to transport synthetic nucleic acids into live tissues to mimic overexpression and interference of a specific PROMPT. The altered expression of *PROMPT_1281* induced the expression of its *cis* and *trans* targets, and this interaction was governed by its secondary structure rather than just its primary sequence. Based on this example, we proposed a model that a concentration gradient of *PROMPT_1281* is established, which increases the probability of its interaction with targets near its transcription site that shares common motifs. Our results firstly demonstrated that *PROMPT_1281* act as carriers of MYB transcription factors to induce the expression of target genes under osmotic stress. In sum, our study identified and validated a set of poplar PROMPTs that likely have regulatory functions in osmotic responses.

## Introduction

Large‐scale RNA sequencing analysis has indicated that more than 90% of eukaryotic genomes are actively transcribed to yield a highly complex network of protein‐coding transcripts and noncoding RNAs (Djebali *et al*., 2012; Hangauer *et al*., 2013). Protein‐coding genes make up only 1%–2% of all transcripts, indicating the widespread occurrence of noncoding RNAs in eukaryotic genomes (Hangauer *et al*., 2013; Kim and Sung, 2012). Functional noncoding RNAs are divided into housekeeping and regulatory RNAs (Chen and Carmichael, 2010; Shuai *et al*., 2014). Based on their extraordinary differences in transcript lengths and biogenesis, classification of regulatory noncoding RNAs remains difficult. Long noncoding RNAs (lncRNAs) are usually classified as RNAs greater than 200 nucleotides (nt) that lack significant protein‐coding capacity (Ulitsky and Bartel, 2013). Depending on their orientation and/or proximity to protein‐coding genes, ncRNAs are annotated as promoter upstream transcripts (PROMPTs), enhancer RNAs (eRNAs), long intervening/intergenic ncRNAs (lincRNAs) and natural antisense transcripts (NATs). Additionally, many lncRNAs are annotated as small nucleolar RNA‐ended lncRNAs (sno‐lncRNAs), 5′snoRNA‐ended and 3′‐polyadenylated lncRNAs (SPAs), circular RNAs (circRNAs) and circular intronic RNAs (ciRNAs) depending on their RNA processing pathways (Wu *et al*., 2017).

LncRNAs are key regulators of gene expression at both the transcriptional and the post‐transcriptional levels in diverse cellular contexts and biological processes (Chen, 2016; Quinn and Chang, 2016). LncRNAs can regulate gene expression in *cis‐* or *trans*‐acting. *Cis*‐acting lncRNAs function near the site of their synthesis and act directly on one or several contiguous genes on the same strand or chromosome. Thus, we speculated that the orientation and/or proximity of lncRNAs to protein‐coding genes might be the main factor for determining whether they act in *cis*. The eRNAs have enhancer‐like functions and can control promoter and enhancer interactions (Li *et al*., 2013; Melo *et al*., 2013). COOLAIR, a NAT transcribed from the *FLOWERING LOCUS C* (*FLC*) gene, mediates the formation of a stable RNA–DNA triplex and an R‐loop (Sun *et al*., 2013a,b; Wahba and Koshland, 2013). The R‐loop recruits a transcription repressor, which results in repression of *FLC*. By contrast, *trans*‐acting lncRNAs diffuse from the site of their synthesis and can act directly on many genes at great distances, even genes on other chromosomes (Lee, 2012). These lncRNA‐mediated interactions might be affected by the structure of the lncRNA. Considering the diversity in biogenesis and biological functions of lncRNAs, the biological function of each lncRNA should be validated, depending on its classification. Up to now, except lincRNAs, NATs and circRNAs, majority of lncRNAs regulatory function is still unclear that might hide some especial transcriptional mechanism of plants.

In mammals, PROMPTs are transcribed in the antisense orientation and from a distance of approximately 0.5–2.5 kb from the transcription start sites (TSSs) of protein‐coding genes (Balbin *et al*., 2015; Preker *et al*., 2008). PROMPTs contain 5′‐cap structures and 3′ adenosine tails and are diversified in length, ranging from 200 to 600 nt (Preker *et al*., 2011). PROMPTs also form complexes with RNA Polymerase II (Pol II) to act on protein‐coding genes (Preker *et al*., 2011). PROMPTs are usually retained in the nucleus and undergo rapid degradation by the RNA nuclear exosome‐targeting complex (Lubas *et al*., 2015; Preker *et al*., 2011). The expression of PROMPTs is cued by environment signals, and their accumulation influences the binding of transcription factors to promoters and is associated with the choice of promoter directionality (Ntini *et al*., 2013). This suggests that, although PROMPTs are short‐lived, they may have important regulatory functions (Lloret‐Llinares *et al*., 2016).

Unlike other lncRNAs, PROMPTs are transcribed from upstream of protein‐coding genes and share many single‐strand *cis* elements with the promoter regions of the neighbouring protein‐coding genes. CREB‐binding protein/E1A‐binding protein p300, a transcription co‐activator, has a unique regulatory motif in which the RNA‐binding region is bound by eRNAs to stimulate histone acetyltransferase activity (Bose *et al*., 2017). These findings suggest that these common *cis* elements might provide potential binding sites for PROMPTs, implying that PROMPTs may be co‐activators for the expression of genes with common transcription factor interaction motifs. However, it is unknown if PROMPTs can induce gene expression in a *trans*‐acting manner.

Higher‐order structures govern most of the functions of lncRNAs, including interactions with proteins, small‐molecule ligands, multicomponent complexes and other RNAs (Dethoff *et al*., 2012; Sharp, 2009). Plants, especially perennial and dioecious plants, have high levels of heterozygosity in their genomes. Abundant single nucleotide polymorphisms (SNPs), insertions/deletions (InDels) and simple sequence repeats in lncRNAs can affect their biological function by changing their secondary structure, altering their stability or interfering with RNA–protein interactions (Ding *et al*., 2012). It is worth noting whether these genetic variants will affect biological function of lncRNA alleles through changing their secondary structure. Differential expression between different lncRNA alleles is highly dependent on cell type or environment stimulus (Bell and Beck, 2009). Even small differences in the level of expression between alleles can strongly affect important physiological processes in mammals, but less is known about the molecular basis of differential expression between alleles in terms of adaptation to distinct developmental processes or different environmental signals.

Here, we systematically identified and characterized osmotic stress‐responsive PROMPTs in poplar at a genomewide scale. We identified SNPs in the PROMPTs, detected their linkage disequilibrium (LD) and dissected the structural variation between the different PROMPT alleles. Then, we analysed the *cis‐* and *trans*‐acting regulatory functions of the PROMPT alleles. We developed a new procedure that uses layered double hydroxides (LDHs), sheet‐like nanoparticles that can transport negatively charged biomolecules into intact plant cells, to deliver RNA molecules to mimic gene overexpression or gene silencing and used this method to validate the functions of a candidate PROMPT. In summary, the results of this study increased our understanding of osmotic stress‐responsive PROMPTs in a perennial plant and provided a new layer for further research on the transcriptional mechanisms of lncRNAs.

## Results

### Identification and characterization of osmotic‐responsive PROMPTs

**Figure 1:**
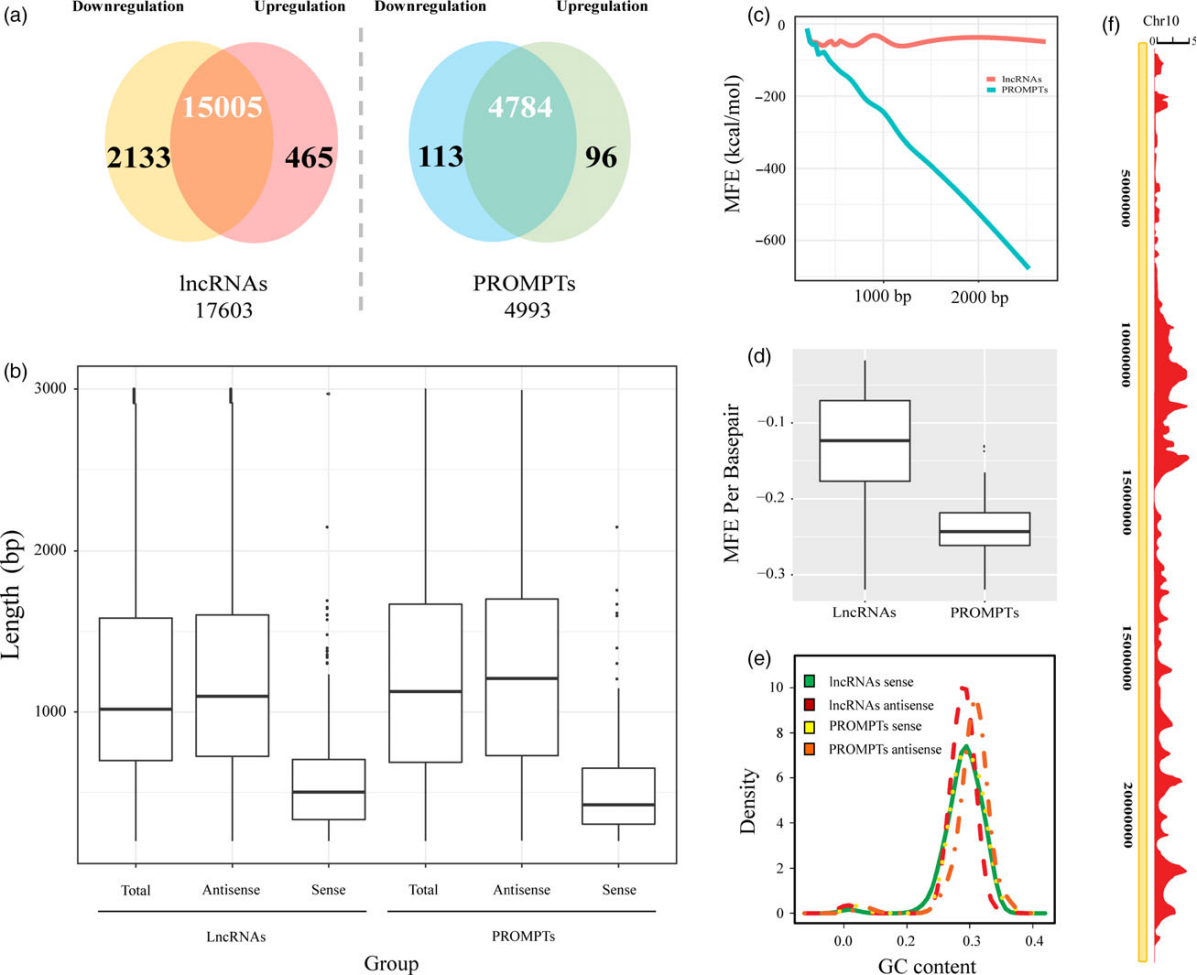
Characteristics of the osmotic stress‐responsive PROMPTs in poplar. (a) Number of lncRNAs and PROMPTs that were differentially expressed in response to osmotic stress. (b) Length distribution of osmotic‐responsive lncRNAs and PROMPTs in sense and antisense strands. (c) Correlation of the minimum free energy (MFE) with the length of the osmotic stress‐responsive lncRNAs and PROMPTs. (d) MFE per base pair of the osmotic stress‐responsive lncRNAs and PROMPTs. (e) GC content density distribution of the osmotic stress‐responsive lncRNAs and PROMPTs that produce transcripts from sense and antisense strands in the poplar genome. (f) Distribution of osmotic stress‐responsive PROMPTs in chromosome 10. The scale shows the mean number of PROMPTs in a 20 kb region.

To identify PROMPTs that are differentially expressed in response to osmotic stress, we conducted genomewide RNA sequencing on control and osmotic‐treated poplar leaves. We obtained approximately 144 and 148 million clean reads from the control and osmotic‐treated groups, respectively. Mapping showed that 78.6% of the reads from the control group and 88.4% from the osmotic‐treated group mapped to the *Populus trichocarpa* genome (Table S1). In total, we obtained 17 603 lncRNAs between both libraries (Figure 1a, Table S2). The lengths of the lncRNAs ranged from 203 to 3002 bp, and most were in the range of 751–1346 bp (Figure 1b). We also obtained 4993 putative PROMPTs between both libraries (Table S3). The lengths of the PROMPTs ranged from 203 to 2902 bp, and most were in the range of 801–1697 bp, which were longer than the average length of the total lncRNAs (Figure 1b). The GC content of the antisense lncRNA loci ranged from 38.4% to 50.3%, which tended to be higher than antisense PROMPTs loci (Figure 1e). The minimum free energy (MFE) of the PROMPTs significantly decreased with increasing length (Figure 1c), and the MFE per bp of the PROMPTs was significantly lower than that of the other lncRNAs (Figure 1d). These results indicated that the structures of the osmotic‐responsive PROMPTs are more stable than those of the other lncRNAs. The distribution of the PROMPTs in the *Populus* chromosomes was examined. In chromosome 10, there were 124.1 PROMPTs within every one Mb, which was the highest density of PROMPTs among all of the chromosomes (Figure 1f). Chromosome 19 had the lowest density, with 46.6 PROMPTs per one Mb (Figure 1f). We also calculated the expression levels of the PROMPTs in fragments per kilobase of transcript per million mapped reads (FPKM), which ranged from 2.9^E−06^ to 1121.9 FPKM (average 33.94 FPKM), which were significantly higher than the expression levels of the other lncRNAs (average 1.64 FPKM) (Table S4).

### Expression of osmotic stress‐responsive PROMPTs

**Figure 2:**
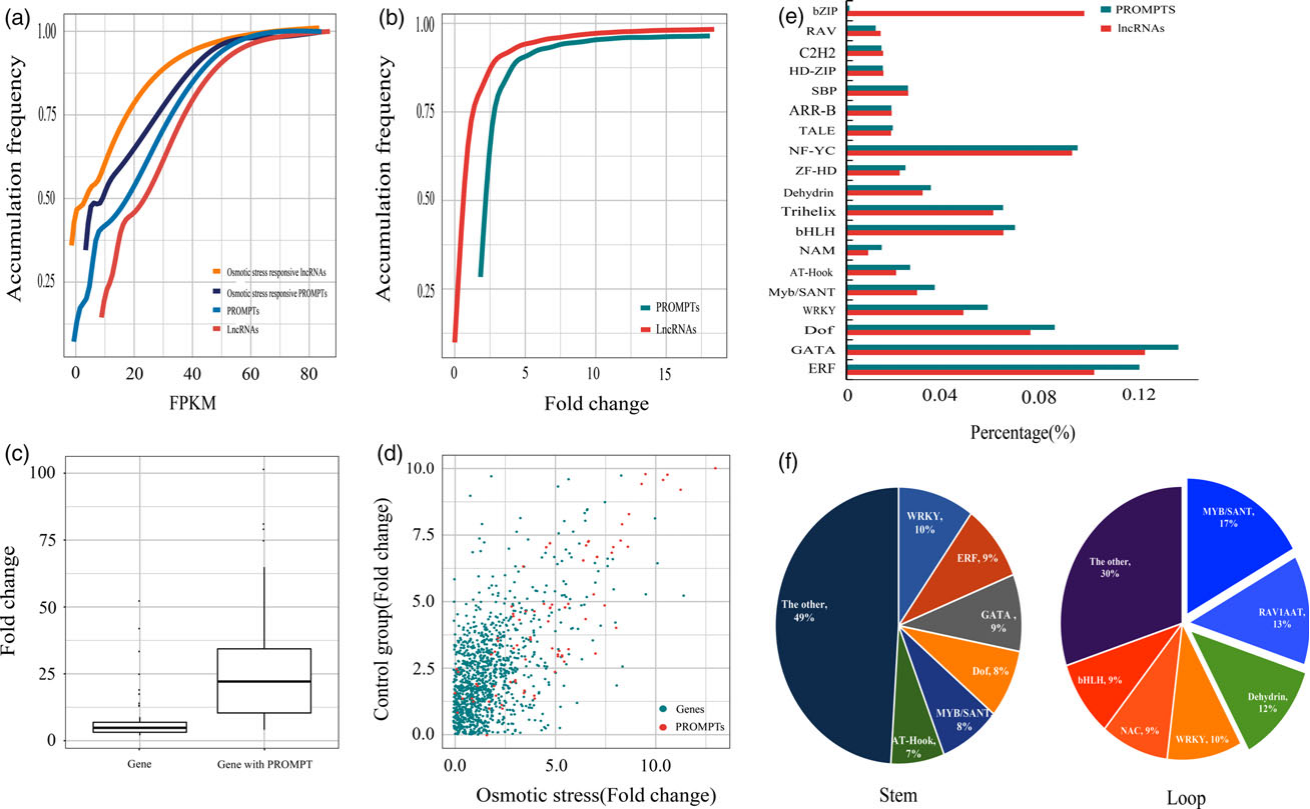
Transcript abundance and motif prediction of the osmotic stress‐responsive lncRNAs and PROMPTs. (a) Accumulation frequency of transcript abundance of lncRNAs and PROMPTs. (b) Accumulation frequency of fold changes of lncRNAs and PROMPTs. (c) Expression patterns of PROMPTs and downstream genes. (d) Correlation of the expression of PROMPTs and downstream genes under osmotic stress. (e) Motif prediction of osmotic stress‐responsive lncRNAs and PROMPTs. (f) Ratio of predicted motifs in the secondary structure of osmotic stress‐responsive PROMPTs.

To identify the potential transcriptional regulatory functions of the PROMPTs, we analysed the transcript abundance of the osmotic stress‐responsive protein‐coding genes and PROMPTs. In total, we identified 2598 lncRNAs that were differently expressed under osmotic stress, including 2133 that were down‐regulated and 465 that were up‐regulated (Figure 1a). The expression levels of the up‐regulated lncRNAs in the osmotic and control groups averaged 1220 and 380 FPKM (average fold change 9.6), respectively (Figure 2a). The expression levels of the down‐regulated lncRNAs in the osmotic and control groups averaged 38 and 141 FPKM (average fold change 0.13), respectively (Figure 2a). We also identified 209 PROMPTs that were differently expressed under osmotic stress (fold change >2 or <0.5, *P *<* *0.005, FDR <0.05), including 113 that were down‐regulated and 96 that were up‐regulated (Table S5). The expression levels of the up‐regulated PROMPTs in the osmotic and control groups averaged 69 and 15 FPKM (average fold change 13.9), respectively (Figure 2c). The expression levels of the down‐regulated PROMPTs in the osmotic and control groups averaged 4.3 and 113 FPKM (average fold change 0.03), respectively. The range of the fold changes of the osmotic stress‐responsive PROMPTs was larger than that of the osmotic stress‐responsive lncRNAs, suggesting that PROMPTs are more responsive to osmotic stress (Figure 2a,b). Protein‐coding genes with osmotic stress‐responsive PROMPTs in their upstream region also had larger fold changes than the other differentially expressed genes (Figure 2c), and the transcript abundance of these genes was positively correlated with the transcript abundance of the PROMPTs in the control and osmotic stress–stress groups (*r *=* *0.65, *P *< 10^−5^) (Figure 2d).

To explore the putative *cis*‐regulatory functions of the osmotic stress‐responsive PROMPTs, we compared the transcript abundance between the osmotic stress‐responsive PROMPTs and their neighbouring genes. The expression of the PROMPTs transcribed from sense and antisense orientations was significantly higher than the expression of downstream protein‐coding genes (Figure S1a,b). The expression of sense/sense pairs and antisense/antisense pairs of PROMPTs and downstream protein‐coding genes was also consistent with the above tendency (Figure S1c,d). Downstream protein‐coding genes were expressed significantly higher than the PROMPTs only when the PROMPTs were transcribed from the opposite direction (Figure S2).

### Motif prediction from the primary sequences of osmotic stress‐responsive PROMPTs

To identify the enriched regulatory elements in the PROMPTs, the motifs present in the promoters of the osmotic stress‐responsive lncRNAs were used as the background. Six motifs, ERF, GATA, Dof, WRKY, MYB/SANT and AT‐Hook, were specifically enriched in the osmotic stress‐responsive PROMPTs and were present in 66.3%–82.6% of the osmotic stress‐responsive PROMPTs (Figure 2e). In addition, bZIP, RAV and C2H2 elements were significantly reduced in the osmotic stress‐responsive PROMPTs. Among these, all motifs bind transcriptional activators, except RAV1AAT, which bind transcriptional repressor. Two ethylene‐responsive elements (RAV1AAT and ERF) were over‐represented in the osmotic stress‐responsive lncRNAs and PROMPTs, respectively, suggesting that osmotic stress‐responsive PROMPTs might participate in an independent ethylene‐responsive regulatory pathway. One abscisic acid (ABA)‐responsive element (ATHB5ATCORE), which acts as a positive regulator of ABA‐responsive genes, was enriched in the PROMPTs. Examination of the distribution of these motifs revealed that MYB/SANT, RAV1AAT and Dehydrin were most enriched in the loop regions of the secondary structure of the PROMPTs (Figure 2f). Scanning of the sequences revealed that 43.8% of the osmotic stress‐responsive PROMPTs contain multiple copies of these motifs. A MYB‐related gene, *Potri.001G219100*, duplicated homeodomain‐like superfamily protein. We found that 26% of the osmotic stress‐responsive PROMPTs contain six copies of the MYB/SANT element and potentially interact with *Potri.001G219100*, implying that these PROMPTs might contain more potential interaction sites for transcription factors (Table S6).

### Co‐expression of osmotic stress‐responsive PROMPTs

To identify potential novel regulators of osmotic stress‐responsive PROMPTs, we constructed a co‐expression network that included 209 osmotic stress‐responsive PROMPTs and 2598 differentially expressed genes. The network connected pairs of genes with high normalized co‐expression (*Z*‐score > 5). PROMPTs were then ranked according to the number of co‐expressed genes in their network cluster. The whole co‐expression network consisted of five independent subnetworks (Figure S3). The two main subnetworks included 96 up‐regulated PROMPTs and 113 down‐regulated PROMPTs. The three other subnetworks contained only one osmotic stress‐responsive PROMPT each. Notably, several of the osmotic stress‐responsive PROMPTs were highly central in the co‐expression network, indicating that they may serve important functional roles in response to osmotic stress in poplar.

**Figure 3:**
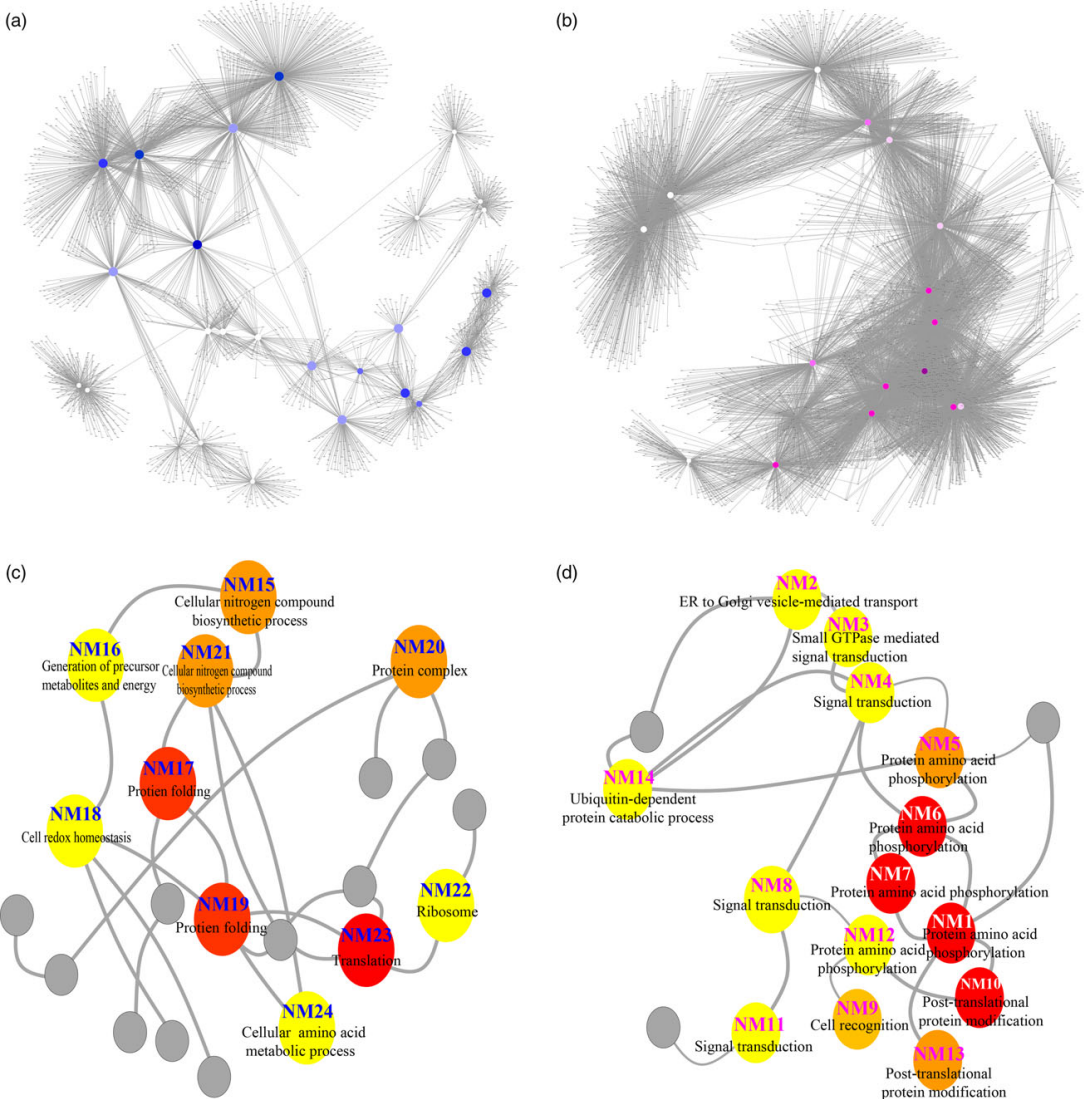
Functional annotation of the osmotic stress‐responsive PROMPTs in the co‐expression network. (a and b) Co‐expression network of osmotic stress‐responsive PROMPTs and protein‐coding genes (correlation coefficient >0.9999, *P *<* *0.0001). Blue nodes represent down‐regulated osmotic stress‐responsive PROMPTs. Red nodes represent up‐regulated osmotic stress‐responsive PROMPTs. (c) Functional annotation of the core node of the down‐regulated co‐expression network. Red nodes represent over 400 protein‐coding genes co‐expressed with PROMPTs. Orange nodes represent over 300 protein‐coding genes co‐expressed with PROMPTs. Yellow nodes represent over 200 protein‐coding genes co‐expressed with PROMPTs. Grey nodes represent PROMPTs with no significant GO enrichment. (d) Functional annotation of the core node of the up‐regulated co‐expression network. Red nodes represent over 800 protein‐coding genes co‐expressed with PROMPTs. Orange nodes represent over 700 protein‐coding genes co‐expressed with PROMPTs. Yellow nodes represent over 600 protein‐coding genes co‐expressed with PROMPTs. Grey nodes represent PROMPTs with no significant GO enrichment.

To categorize the biological processes transcriptionally regulated by the osmotic stress‐induced PROMPTs, we utilized the co‐expression network to identify representative network modules (NMs) containing nonoverlapping sets of genes that were highly co‐expressed with the most central genes in the network (Figure 3). Using Gene Ontology (GO) enrichment analyses, we assigned putative biological functions to the 27 main NMs (including 15 up‐regulated and 12 down‐regulated osmotic stress‐responsive PROMPTs) containing at least 51 co‐expressed genes (Figure 3 and Table 1). For the down‐regulated osmotic stress‐responsive PROMPTs, each node had an average of 135.9 co‐expressed genes; for the up‐regulated osmotic stress‐responsive PROMPTs, the average was 469.6 co‐expressed genes, which was significantly higher. We observed co‐expressed genes significantly enriched within NMs. NM1 (*PROMPT_1281*, protein amino acid phosphorylation, *P *<* *5.65^E−21^; cell recognition, *P *<* *2.57^E−07^) contained 838 genes associated with protein phosphorylation and cell signalling, including seven homologs of *WALL‐ASSOCIATED KINASE* (*WAKL*) and three homologs of *MYB*. This module was also enriched in phytohormone‐related genes, including homologs of *ETHYLENE RESPONSE FACTOR 1* (*ERF1*), *CYTOKININ OXIDASE 6* (*CKX6*) and auxin‐induced proteins, which are involved in the phytohormone‐activated signalling pathway. Genes in this module were significantly down‐regulated upon osmotic stress, suggesting negative roles in response to osmotic stress (Figure 3d). NM25 (*PROMPT_3649*, photosynthesis, *P *<* *1.87^E−16^) was enriched in photosynthesis‐related genes and included *PHOTOSYSTEM I SUBUNIT F* (*PSAF*), *LIGHT HARVESTING COMPLEX PHOTOSYSTEM II SUBUNIT 6* (*LHCB6*), *PROTOCHLOROPHYLLIDE OXIDOREDUCTASE A* (*PORA*) and *PHOTOSYSTEM II REACTION CENTER W* (*PSBW*), which have a demonstrated role in light reactions and photorespiration (Figure S3, Table S7). This module also included *MYB4* and *CTL* genes. *MYB4* gene is a well‐known negative regulator of transcript (Zhao *et al*., 2007). *CTL* which encodes an endo‐chitinase‐like protein is essential for tolerance to heat, salt and osmotic stresses (Hong *et al*., 2003).

**Table 1:** Annotation of the co‐expression network modules

Network module	PROMPT	Expression pattern	Enrichment GO term	Annotation
NM1	*PROMPT_1281*	Up‐regulated	GO:0006468	Protein amino acid phosphorylation
NM2	*PROMPT_3076*	Up‐regulated	GO:0006888	ER to Golgi vesicle‐mediated transport
NM3	*PROMPT_4986*	Up‐regulated	GO:0007264	Small GTPase‐mediated signal transduction
NM4	*PROMPT_1280*	Up‐regulated	GO:0007165	Signal transduction
NM5	*PROMPT_0386*	Up‐regulated	GO:0006468	Protein amino acid phosphorylation
NM6	*PROMPT_2924*	Up‐regulated	GO:0006468	Protein amino acid phosphorylation
NM7	*PROMPT_2536*	Up‐regulated	GO:0006468	Protein amino acid phosphorylation
NM8	*PROMPT_1393*	Up‐regulated	GO:0007165	Signal transduction
NM9	*PROMPT_0270*	Up‐regulated	GO:0008037	Cell recognition
NM10	*PROMPT_4201*	Up‐regulated	GO:0043687	Post‐translational protein modification
NM11	*PROMPT_0293*	Up‐regulated	GO:0007165	Signal transduction
NM12	*PROMPT_1714*	Up‐regulated	GO:0006468	Protein amino acid phosphorylation
NM13	*PROMPT_4695*	Up‐regulated	GO:0043687	Post‐translational protein modification
NM14	*PROMPT_4653*	Up‐regulated	GO:0006511	Ubiquitin‐dependent protein catabolic process
NM15	*PROMPT_0982*	Down‐regulated	GO:0034641	Cellular nitrogen compound biosynthetic process
NM16	*PROMPT_1524*	Down‐regulated	GO:006091	Generation of precursor metabolites and energy
NM17	*PROMPT_4428*	Down‐regulated	GO:0006457	Protein folding
NM18	*PROMPT_0317*	Down‐regulated	GO:0045454	Cell redox homeostasis
NM19	*PROMPT_2181*	Down‐regulated	GO:0006457	Protein folding
NM20	*PROMPT_1259*	Down‐regulated	GO:0043234	Protein complex
NM21	*PROMPT_1588*	Down‐regulated	GO:0034641	Cellular nitrogen compound biosynthetic process
NM22	*PROMPT_3520*	Down‐regulated	GO:0005840	Ribosome
NM23	*PROMPT_0258*	Down‐regulated	GO:0006412	Translation
NM24	*PROMPT_1220*	Down‐regulated	GO:0006520	Cellular amino acid metabolic process
NM25	*PROMPT_3649*	Up‐regulated	GO:0015979	Photosynthesis
NM26	*PROMPT_2774*	Down‐regulated	GO:0006091	Generation of precursor metabolites and energy
NM27	*PROMPT_3662*	Down‐regulated	None	None

### Effect of nucleotide variations in the secondary structure of osmotic stress‐responsive PROMPTs

**Figure 4:**
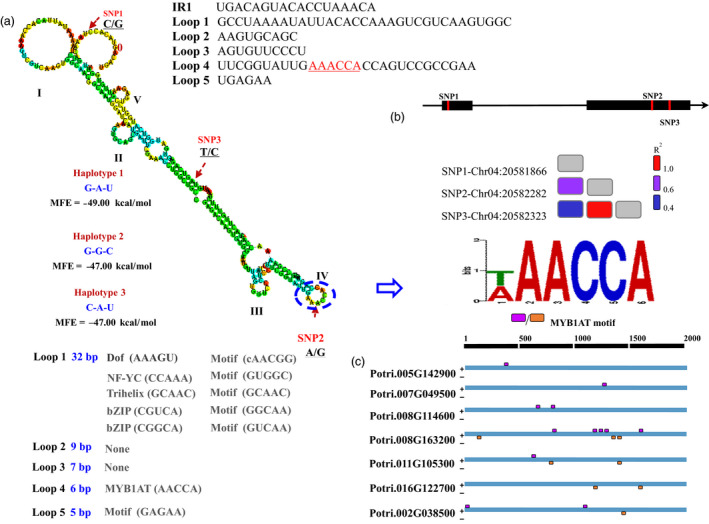
Secondary structure and motif prediction of *PROMPT_1281*. (a) Schematic diagram of the secondary structure and sequences of *PROMPT_1281*. I–V represents the five loops which might be potential interaction regions for binding with RNA‐binding proteins. Loops 1–5 represent motif annotation in the sequence of those loops. SNP1–SNP3 represent single nucleotide polymorphisms in the natural population of poplar. (b) Linkage disequilibrium of the PROMPT‐SNPs (*R* ^2^ > 0.6). (c) Distribution of the MYB1AT motif which is located in loop 4 in *trans* targets, including *Potri.005G142900*,* Potri.007G049500*,* Potri.008G114600*,* Potri.008G163200*,* Potri.011G105300*,* Potri.016G122700* and *Potri.002G038500*. The pink square represents the motif located in sense strands. The orange square represents the motif located in antisense strands.

To investigate the function of the osmotic stress‐responsive PROMPTs, we chose *PROMPT_1281* (NM1) for further study, as the NM1 PROMPT_1281 node had the most connection with the other nodes in the co‐expression network. Eight SNPs were found in the genomic regions of *PROMPT_1281*, including three SNPs in two exons and five SNPs in an intron (Figure 4). To examine the effects of the SNPs on the secondary structure of the RNA, we predicted the secondary structures of the different *PROMPT_1281* variants and calculated the MFE. The average energy change due to the SNPs was 0.8 kcal/mol and ranged from 0 to 2 kcal/mol, which suggested that the SNPs have significant effects on the secondary structure.

To assess the overall behaviour of LD within the SNPs in *PROMPT_1281*, we calculated the *R* ^2^ values. Figure 4 shows a larger number of SNPs that were in linkage equilibrium (*r* ^2^ < 0.3; *P *<* *0.001) across the sequenced regions. Only one pairwise (SNP 04G20582282‐SNP 04G20582323) interaction showed significant linkage disequilibrium in the candidate PROMPT (*r* ^2^ = 0.9; *P *<* *0.0001). Additionally, we found three haplotypes of *PROMPT_1281* in a natural population consisting of 505 individuals. Among these, haplotype 1 (Hap1) and haplotype 2 (Hap2) were the most abundant, consisting of 59.5% and 39.4% of all haplotype, respectively (Figure 4). These two predominant haplotypes had the same secondary structure, except that the MFE of Hap1 (−49.00 kcal/mol) was lower than that of Hap2 (−47.00 kcal/mol) (Figure S4).

To identify the potential function of the loop structure of *PROMPT_1281*, we separately scanned the potential transcription factor binding motif in the stem and loop sequences. The predicted motif was significantly enriched in the loop regions (*P *<* *0.0001), including MYB/SANT, bZIP, Dof, C2H2 and Trihelix. This characteristic was validated in all osmotic stress‐responsive PROMPTs and indicated that nucleotide variations might affect the regulatory functions of PROMPTs through alterations in their secondary structure.

To evaluate whether these osmotic stress‐responsive PROMPTs were under natural selection, we compared the nucleotide substitution rate (*d*_PROMPT_) to the equivalent rate (*d*_AR_) within neighbouring ancestral repeats (ARs). This ratio is analogous to the ratio of nonsynonymous to synonymous substitution rates in a protein‐coding sequence. The values of *d*_PROMPT_ estimated between the poplar PROMPT sequences aligned to their *Arabidopsis thaliana* orthologous sequences were significantly lower than those of *d*_AR_ (*P *< 10^−6^). Median *d*_lncRNA_/*d*_AR_ values for the lncRNAs were 0.931 (*Populus*–*Arabidopsis*) (Figure S5). This indicated that SNPs in osmotic stress‐responsive lncRNAs undergo natural selection.

### Allelic expression pattern of *PROMPT_1281*

**Figure 5:**
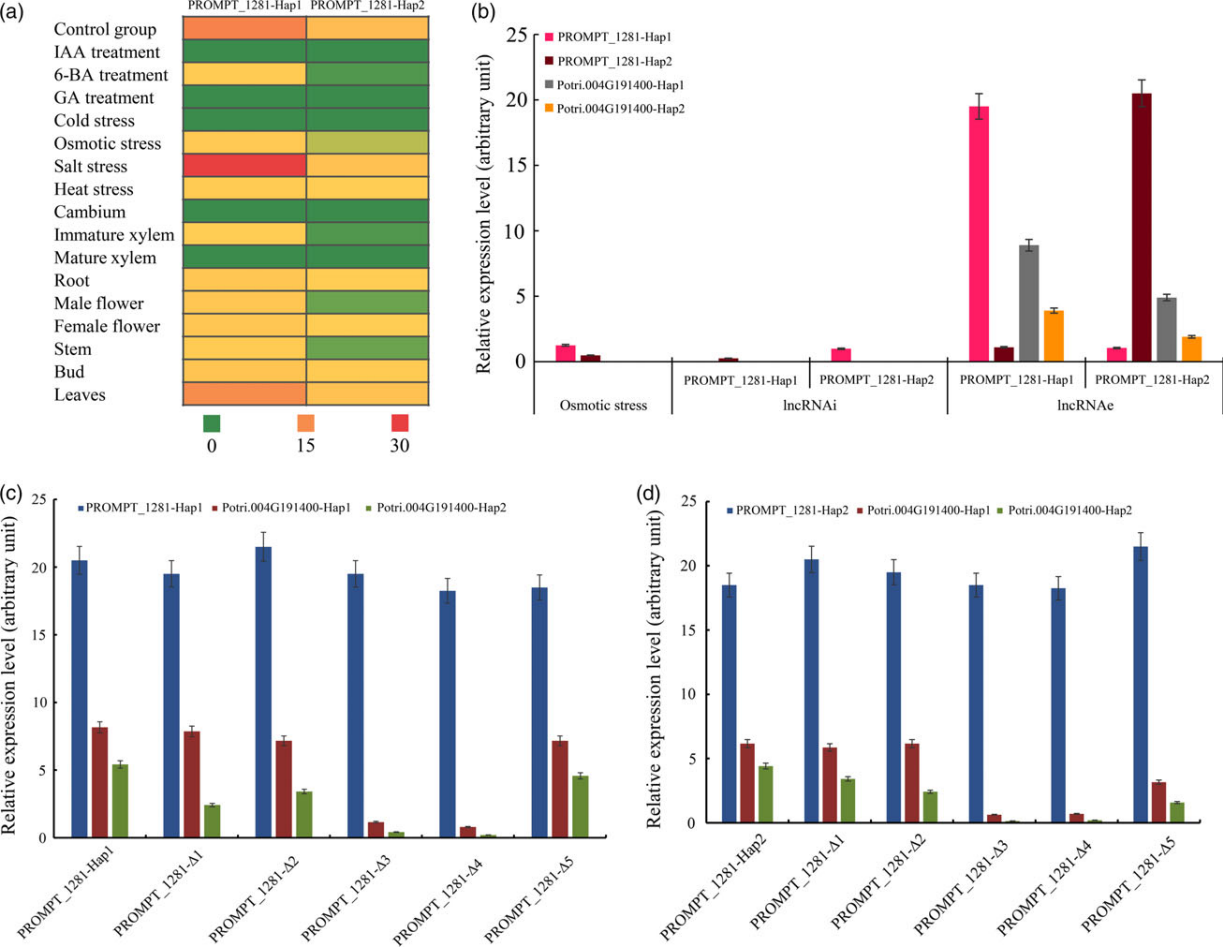
Allelic expression pattern of *PROMPT_1281* and its *cis* targets. (a) Allelic expression pattern of *PROMPT_1281* in different tissues and abiotic stresses. (b) Allelic expression pattern of *PROMPT_1281* and its *cis* targets under lncRNAi and lncRNAe treatments. (c) Allelic expression pattern of *Potri.004G191400* under overexpression of *PROMPT_1281‐Hap1* with a mutation in its secondary structure. (d) Allelic expression pattern of *Potri.004G191400* under overexpression of *PROMPT_1281‐Hap2* with a mutation in its secondary structure. Relative transcript levels were calculated by qPCR with *ACTIN* as the standard. Data are mean ± SE of three separate measurements. Error bars represent standard error.

To profile the allelic expression pattern of *PROMPT_1281*, we used seven tissue samples, four abiotic stress‐treated samples and three phytohormone‐treated samples for quantitative PCR (qPCR) analysis. We cloned full‐length cDNAs of *PROMPT_1281*. Sequence analysis revealed several alleles, *PROMPT_1281‐Hap1* and *PROMPT_1281‐Hap2*. As shown in Figure 5, the candidate genes exhibited different expression patterns. Transcript abundances of *PROMPT_1281‐Hap1* were significantly higher than those of *PROMPT_1281‐Hap2*, indicating imbalanced expression of the different *PROMPT_1281* alleles. The expression specificity value (tau score) of *PROMPT_1281‐Hap1* was 0.93, which was significantly higher than that of *PROMPT_1281‐Hap2*. Among the nine tissues, both alleles of *PROMPT_1281* were expressed except in cambium and mature xylem. The expression of *PROMPT_1281‐Hap1* was significantly higher than that of *PROMPT_1281‐Hap2* in leaves, male flowers and roots. The expression of *PROMPT_1281‐Hap2* was higher than that of *PROMPT_1281‐Hap1* only in immature xylem. For the different abiotic stress treatments, the two alleles were significantly up‐regulated under salt stress and significantly down‐regulated under osmotic and heat stress. Neither of the alleles was expressed under cold stress. For the phytohormone treatments, neither of the alleles was expressed under the gibberellin (GA) and auxin (IAA) treatments, but both were significantly down‐regulated under cytokinin (6‐BA) treatment.

### *PROMPT_1281* is a positive regulator of *PsiWALL‐ASSOCIATED KINASE 2*

*PROMPT_1281* is transcribed 200 bp upstream of the TSS of *PsiWALL‐ASSOCIATED KINASE 2* (*PsiWAK2*,* Potri.004G191400*). *PROMPT_1281* and *PsiWAK2* start transcription in the same direction. Therefore, we assumed that *PROMPT_1281* might target *PsiWAK2* in a *cis*‐acting manner. Sequence analysis showed heterozygous allele of *PsiWAK2*, marking as *PsiWAK2*‐*Hap1* and *PsiWAK2*‐*Hap2*, respectively. The two *PsiWAK2* alleles were highly expressed in the control group but significantly down‐regulated with a concurrent decrease in *PROMPT_1281* transcripts under osmotic stress. To detect the relationship of *PROMPT_1281* and its targets, lncRNAi and lncRNAe analyses (Figure S6) were used to mimic the RNA silencing and overexpression effect, respectively. When *PROMPT_1281* was silenced, *PsiWAK2*‐*Hap1* and *PsiWAK2*‐*Hap2* were significantly down‐regulated compared with the control group. When abundance of *PROMPT_1281* was increased, *PsiWAK2*‐*Hap1* and *PsiWAK2*‐*Hap2* were significantly up‐regulated in the control group and under osmotic stress. Therefore, we speculated that *PROMPT_1281* might function as a positive regulator of its targets.

Our study showed that the predicted transcription factor binding motif is significantly enriched in the loop regions, we assumed that these loop structures might play important roles in transcriptional regulation. To test the molecular function of the secondary structure of *PROMPT_1281*, we deleted the five loop sequences from *PROMPT_1281* one by one. *In silico* RNA structure analysis revealed that, when loops 1–5 were deleted, the MFE increased from 2.2 to 5 kcal/mol, suggesting that these loops might play an important role in maintaining the stability of the secondary structure. All five secondary structure mutant sequences were synthesized for subsequent lncRNAe experiments. *PROMPT_1281* mutants lacking loop 3 and loop 4 were not able to significantly induce the expression of the two alleles of its *cis*‐target *PsiWAK2*, suggesting that loops 3 and 4 are required for its *cis*‐regulatory function.

To detect the interacting regions of the PROMPTs and candidate promoters, we used several criteria to scan the candidate regions. First, the length of the interacting regions must be sufficient to bind specific sequences. Second, unwinding of the interacting regions should not change the whole secondary structure of the PROMPTs. Based on these two criteria, we found one potential candidate region (Interaction Region 1, IR1) after whole‐sequence scanning (Figure 4). After that, we deleted IR1 in *PROMPT_1281* to ensure it could not pair with potential interacting regions (Table S8). LncRNAe analysis showed that the transcript abundance of the two alleles of its *cis*‐target was not significantly altered after interaction with the *PROMPT_1281* mutant lacking IR1. This result implied that *PROMPT_1281* might not interact with the promoter of its *cis* targets.

### *PROMPT_1281* activates its co‐expressed genes

We identified six genes that are co‐expressed with *PROMPT_1281* using strict criteria (correlation coefficient >0.9999, *P *<* *0.0001). GO analysis was used on these protein‐coding genes to examine their potential functions. The results revealed that 17 GO terms were enriched in these genes, including protein amino acid phosphorylation, post‐translational protein modification and protein modification processes (Table S9). This result indicated that *PROMPT_1281* might be an activator in signal transduction. Sequences analysis showed that the MYB1AT motif is enriched in the promoter of six co‐expressed genes (confidence = 100). The MYB1AT motif is a MYB recognition site found in the promoter of the dehydration‐responsive gene *RD22* in Arabidopsis (Abe *et al*., 2003). This suggested that the MYB1AT motif might be a corecognition site in co‐expressed genes. *MYB14* was among the six co‐expressed genes, implying that *PROMPT_1281* might have feedback regulation in the MYB transcript factor family.

The six co‐expressed genes and *PROMPT_1281* were significantly down‐regulated under osmotic stress. To investigate the *trans*‐regulation functions of *PROMPT_1281*, lncRNAe analyses were used to mimic the effect of *PROMPT_1281* overexpression on the co‐expressed genes. When abundance of *PROMPT_1281* was increased, the six co‐expressed genes were significantly up‐regulated under osmotic stress, and the transcript abundance of the co‐expressed genes under overexpression of *PROMPT_1281‐Hap1* was significantly higher than that under overexpression of *PROMPT_1281‐Hap2*. Therefore, we speculated that *PROMPT_1281* might also function as an activator of its *trans* targets, and *PROMPT_1281‐Hap1* has stronger activation activity than *PROMPT_1281‐Hap2*.

**Figure 6:**
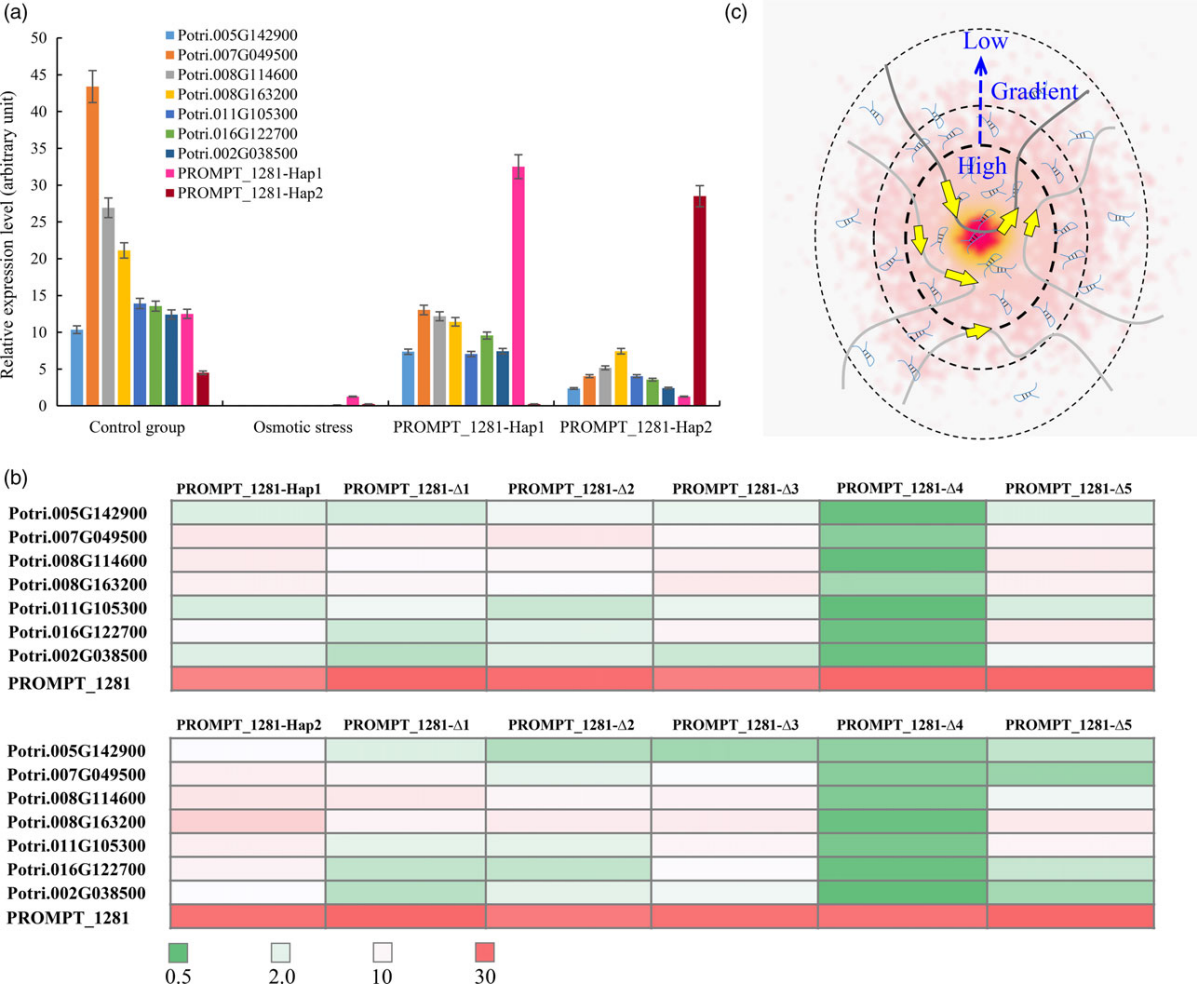
Allelic expression pattern of *PROMPT_1281* and its *trans* targets. (a) Allelic expression pattern of *PROMPT_1281* and its *trans* targets under osmotic stress and lncRNAe treatment. Relative transcript levels were calculated by qPCR with *ACTIN* as the standard. Data are mean ± SE of three separate measurements. Error bars represent standard error. (b) Allelic expression pattern of *trans* targets under overexpression of the *PROMPT_1281* secondary structure mutant. ∆1–∆5 represents deletions of loops 1–5. (c) Schematic diagram of how PROMPTs regulate their *trans* targets’ transcript abundance via a concentration gradient. The concentration of a PROMPT will be highest (red—inner circle) near its site of transcription and will decrease (pink—outer circles) the further the distance from its site of transcription, creating a concentration gradient. This concentration gradient establishes a nuclear domain with a high concentration of the PROMPT, where it can interact with site‐specific targets.

To test the regulatory functions of *PROMPT_1281* via its secondary structure, we also deleted the five loop sequences from *PROMPT_1281* one by one (Figure 6). All five secondary structure mutant sequences were synthesized for subsequent lncRNAe experiments. The results revealed that, without loop 4, neither of the *PROMPT_1281* alleles could significantly induce the expression of the co‐expressed genes, suggesting that only loop 4 is required for the *trans*‐regulation function of *PROMPT_1281*. Additionally, the MYB1AT motif was found in loop 4, implying that the *trans*‐regulation function of *PROMPT_1281* is dependent on this MYB recognition site. To further validate the role of loop 4 in transcriptional regulation, we created mutants by changing three nucleotides in loop 4 to change the secondary structure to a stem (Figure S7). LncRNAe analysis showed that the transcript abundance of the co‐expressed genes was not significantly changed after interaction with the *PROMPT_1281* mutant lacking loop 4 (Figure S8). This result suggested that both the secondary structure and sequence motifs are responsible for the transcriptional regulation functions of *PROMPT_1281*.

## Discussion

### PROMPTs are more stable than lncRNAs

In mammals, PROMPTs are transcribed in the antisense orientation (Balbin *et al*., 2015). Our results showed that approximately 4.5% of PROMPTs are transcribed in the sense orientation in poplar, indicating that PROMPTs are transcribed bidirectionally in plants. The distance of PROMPTs upstream of the active TSSs of most protein‐coding genes ranges from 0.5 to 2.5 kb in mammals (Preker *et al*., 2008). In our study, PROMPTs were found approximately 0.4–0.8 kb upstream of the TSSs of protein‐coding genes, implying that PROMPTs might share a common promoter with downstream genes, resulting in robust *cis*‐mediated transcriptional regulation. Generally, PROMPTs are heterologous in length (about 200–600 nt) in mammals (Preker *et al*., 2011). By contrast, the length of PROMPTs ranged from 0.2 to 2.7 kb in poplar, indicating that the length of PROMPTs might be longer in plants.

Higher GC content confers higher thermostability in DNA and RNA. PROMPTs have higher GC content than other lncRNAs, indicating that PROMPTs might be unique among lncRNAs. CpG islands are regions with a high frequency of CpG sites that are always associated with the TSS of genes (Hartl and Jones, 2005). Because of the proximity of PROMPTs to TSSs, they overlap with CpG islands, which might be the main reason for their higher GC content. PROMPTs were found to be enriched in different chromosomes from other lncRNAs, suggesting that they might be involved in different transcriptional events. A low MFE means that the RNA has a more stable secondary structure (Mathews *et al*., 2004). Our results showed that the MFE of PROMPTs is significantly lower than that of other lncRNAs, suggesting that PROMPTs are more stable than other kinds of lncRNAs. Taken together, our results suggest that PROMPTs have bidirectional transcription in plants, and compared to other lncRNAs, they are longer in length, have a higher GC content and greater stability and are closer in proximity to protein‐coding genes.

### Osmotic stress‐responsive PROMPTs are more sensitive to osmotic stress than other lncRNAs

Previous study reported that lncRNAs are expressed at significantly lower levels in plants and animals (Cabili *et al*., 2011; Shuai *et al*., 2014). In this study, our results showed that PROMPTs were expressed at about twofold higher levels, on average, than other lncRNAs. Osmotic‐responsive PROMPTs were expressed about fourfold higher, on average, than other lncRNAs, and the average fold change of PROMPTs was also higher than that of other lncRNAs, suggesting that PROMPTs are more responsive than other lncRNAs to osmotic stress. In our study, the transcript abundance and fold changes of the genes located downstream of PROMPTs were positively correlated with the expression of the PROMPTs in the control and osmotic stress group, suggesting that PROMPTs might be positive transcriptional regulators of their *cis* targets. When PROMPTs and their gene targets were located in the same transcriptional orientation (sense/sense pairs and antisense/antisense pairs of PROMPTs/downstream protein‐coding genes), the expression level of the PROMPTs was higher than when in the reverse orientation (sense/antisense pairs), implying that their orientation might also play an important role in their regulatory functions.

Sharing of regulatory elements in divergently transcribed genes is a primary factor for their co‐expression (Williams and Bowles, 2004). In our study, the expression of PROMPTs was positively correlated with the expression of neighbouring genes that shared similar motifs, implying that these common motifs might be important regulation sites for the expression of PROMPT‐mRNA pairs. *Cis*‐regulatory elements in the promoter regions of sense and antisense transcripts play an important role in expression patterns (Williams and Bowles, 2004). A large percentage of lncRNAs physically interact with various chromatin regulatory proteins, including PRC2, WDR5 and other proteins involved in chromatin modifications (Guttman *et al*., 2011; Quinodoz and Guttman, 2014). These examples highlight how lncRNAs interact with proteins using their single‐strand sequence. Our results showed that ERF, MYB, CBF/DREB and other motifs are enriched in PROMPTs and might provide potential target sites for transcriptional regulation factors.

### The secondary structure of PROMPTs is more conserved than that of other lncRNAs

With the exception of the transcription factor binding site, the secondary structure of lnRNAs has critical roles in diversified processes including ligand sensing to the regulation of translation, polyadenylation and splicing (Cruz and Westhof, 2009). We predicted and characterized the conserved secondary structure of osmotic stress‐responsive lncRNAs within the same NMs. All five PROMPTs in NM1, which was the largest NM among the co‐expression network, had one conserved secondary structure that was annotated as being related to the regulation of transcription. The loop sequence of this conserved secondary structure contained a transcription factor binding site (a MYB1AT motif). This suggested that the expression pattern of the PROMPTs in NM1 might be mediated by the MYB1AT motif within their conserved secondary structure.

Single nucleotide polymorphisms are thought to be the most widespread factor affecting the structural variations, stability and transcript abundance of RNA (Ding *et al*., 2012; Gong *et al*., 2015). Our results showed that the density of SNPs in PROMPTs was lower than that of other lncRNAs. These SNPs may influence the stability, expression and functions of PROMPTs via changes in their secondary structure. Our analysis of the MFE of the PROMPTs showed that the average energy changes conferred by SNPs in poplar were 2.61 kcal/mol, which is higher than that in other lncRNAs in poplar and mammals, suggesting that SNPs might have more significant effects on the secondary structure of PROMPTs in plants. Linkage disequilibrium analysis showed that the number of PROMPT‐SNPs in LD was significantly larger than that of lncRNA‐SNPs, and most of the LD regions had conserved secondary structures. This suggests that the secondary structure of PROMPTs is more conserved than that in other lncRNAs, which might help maintain stable regulatory functions.

To better understand the functionality of lncRNAs, we investigated whether lncRNAs exhibit signatures of purifying selection. ARs present in the last common ancestor appear to have evolved neutrally (Lunter *et al*., 2006). Their evolutionary rates provide appropriate proxies for the mutational rates in selectively neutral sequences (Ponjavic *et al*., 2007). Under neutrality, the length of intergap segments distributed similarly to distribution predicted for the distance between successive indels. Our Blast Z *Populus*–*Arabidopsis* alignments results showed a remarkably close fit to the geometric distribution (Song, unpublished data). Thus, we compared the estimated rate of nucleotide substitutions (*d*_*PROMPTs*_) to the equivalent rate (*d*_AR_) within neighbouring ARs (*Populus*–*Arabidopsis*). If *d*_*PROMPTs*_/*d*_*AR*_ is equal to one, this would indicate that PROMPT‐SNPs have not experienced selection. If this ratio is significantly less than 1, this would indicate either purifying selection or lower mutation rates. The median *d*_*PROMPTs*_/*d*_*AR*_ values for the PROMPTs were 0.931, indicating that osmotic stress‐responsive PROMPT‐SNPs have undergone purifying selection, and the substitution rates were suppressed by approximately 7%. As this ratio was higher for the PROMPTs than for other lncRNAs, this indicated that PROMPTs undergo stronger purifying selection than other lncRNAs.

### *PROMPT_1281* might be a carrier for regulatory factors that regulate transcription networks

Allele‐specific expression, which has classically been associated with epigenetic phenomena, is essential for normal development and many cellular processes (Bell and Beck, 2009; Knight, 2004). Allele‐specific expression is relatively common among nonimprinted genes (Brem *et al*., 2002; Cheung *et al*., 2003; Enard *et al*., 2002). In foetal liver or kidney tissue, 54% genes showed at least a twofold difference in transcript abundance between alleles in at least one individual, whereas 28% of genes showed a greater than fourfold difference (Knight, 2004). Genomewide disease association studies showed that only a small minority of disease‐associated SNPs are found in protein‐coding gene sequences. Most of the disease‐associated SNPs were found within noncoding intronic or intergenic regions. These results indicated that allele‐specific expression might be due to upstream regulatory sequence variations through *cis*‐acting mechanisms (Oleksiak *et al*., 2002). In our study, *PROMPT_1281*, which is a core regulator in NM1, was selected for allele‐specific expression analysis. The alleles of *PROMPT_1281* showed significantly different expression patterns resulting in different levels of expression of its downstream genes. This indicated that allele‐specific differences in the expression levels of PROMPTs might support a model whereby *cis*‐acting genetic variation results in differential expression between alleles.

Specific secondary structures of lncRNAs, which might be essential for their effect on the DNA‐binding activity of transcription factors by modifying transcription factor dimerization or trimerization (Willingham *et al*., 2005), promoting transcription factor phosphorylation (Wang *et al*., 2014) or controlling transcription factor nuclear localization (Lai *et al*., 2013). The alleles of *PROMPT_1281* have the same secondary structures but significantly different MFE, suggesting that the regulatory functions of the alleles may be limited by their stability. Expression analysis indicated that the secondary structure of *PROMPT_1281* was significantly changed without loops 3 and 4, and these structural mutants could not induce the expression of downstream genes. This finding indicated that loops 3 and 4 are integral to its *cis*‐acting regulatory function. We also found that the *PROMPT_1281* mutant lacking loop 4 was unable to induce the expression of its *trans* target genes, implying that loop 4 is integral for the *trans*‐acting regulatory function of *PROMPT_1281*.

We changed loop 4 of *PROMPT_1281* into a stem structure by mutating the sequence of the complementary strands of the MYB1AT motif. *PROMPT_1281* with mutated loop 4 was not as effective at inducing gene expression as the normal *PROMPT_1281*, suggesting that a specific feature might be required for the transcriptional regulatory functions of PROMPTs. Conserved motifs are essential for RNA to be bound and regulated by RNA‐binding proteins (Ray *et al*., 2013). In our study, even when the primary sequences of the MYB1AT motif were not changed, specific secondary structures were essential for PROMPTs to be bound by RNA‐binding proteins. This suggested that the stability of the secondary structure is important for maintaining its function and might explain why LD was detected in this region.

Our study showed that lncRNAs utilize their secondary structure to bind with upstream target sequences and regulate their transcription (Song, unpublished data). In the current study, *in vitro* experiments showed that *PROMPT_1281* could not hybridize with the promoter sequence of downstream genes, which suggested that *PROMPT_1281* might not interact with DNA for transcriptional regulation of downstream genes. Interestingly, the concentration of *PROMPT_1281* decreased with increasing distance from its site of transcription, creating a concentration gradient. *PROMPT_1281* has a MYB transcription factor binding site (MYB1AT motif) that binds the MYB1 transcription factor (Yadav *et al*., 2017). In cotton, MYB1 regulates a specialized subcomponent of the secondary cell wall involving secondary metabolite synthesis and stress hormone signalling‐related gene networks. Our results showed that *PROMPT_1281* might be a carrier for MYB1 and other regulatory factors that regulate transcription networks. Additionally, *PROMPT_1281* might undergo rapid degradation by the RNA nuclear exosome‐targeting complex (Taft *et al*., 2009). Therefore, the concentration gradient of *PROMPT_1281* might rapidly increase the probability of an interaction of its *cis* and *trans* targets with the MYB transcription factor.

## Experimental procedures

### Plant materials and treatments

One‐year‐old *Populus simonii* ‘QL9’ clones were grown in pots with inner size of 10 cm in height and 15 cm in diameter, containing a potting mix of a commercial medium and perlite at a ratio of 3 : 1. Those clones were maintained under natural light (1250 μmol/m^2^/s of photosynthetically active radiation), 25 ± 1 °C, 50% ± 1 relative humidity and a 12/12‐h day/night regime in an air‐conditioned greenhouse. Relative leaf water content was measured as Schonfeld's described (Schonfeld *et al*., 1988). Relative leaf water content was significantly decreased at 6‐h osmotic stress (Figure S9), implying there might be a substantial change in gene expression at this time point. Therefore, we choose 6‐h osmotic stress treatment for transcriptome analysis.

One‐year‐old clones of the same size (50 cm in height) were used for abiotic stress treatment. These groups were exposed to 150 mm NaCl, 30% polyethylene glycol (PEG) 6000, 42 and 4 °C for 6 h treated for salinity, osmotic, heat and cold stress treatments, respectively. Clones not exposed to abiotic stress were used as the control group (Song *et al*., 2014). For phytohormone treatment, 100 μm of GA_3_, IAA and 6‐BA (Sigma‐Aldrich, St. Louis, MO, USA) were, respectively, sprayed on clone leaves until drops of liquid dripped down. The control plants were treated with water in the same manner. Considering effect of the developmental stages of leaves on gene expression, mature leaves with developmental stages which from the same position of control and treated plants were collected at 6 h after treatment. Three biological replicates were used in each treatment, including the control group. Fresh leaves were collected from all these groups, immediately frozen in liquid nitrogen and stored at −80 °C until analysed. For tissue‐specific gene expression analysis, cambium, immature xylem, mature xylem, root, stem and bud were also collected from 1‐year‐old clones. Male and female flower were collected from 30‐year‐old male and female poplar, respectively. All these tissues also immediately frozen in liquid nitrogen and stored at −80 °C until analysed.

### Sequencing of lncRNA

After the osmotic stress treatment, total RNA was isolated from fresh leaves by a modified CTAB method and was used for small RNA library construction. For lncRNA sequencing, a strand‐specific cDNA library was constructed using the SMART library construction method (Levin *et al*., 2010). The detailed library construction process is presented in the supplemental data. LncRNAs were sequenced using an Illumina HiSeq 2000 at the Shanghai Bio Institute (Shanghai,China). The total number of reads and mapping results is shown in Table S1. The gene expression data reported here are available from NCBI with the SRA database accession numbers SRR5127346.

### Prediction of PROMPTs from the cDNA sequences

Clean reads were obtained after filtering out low‐quality reads and trimming the adaptor sequences. The *P. trichocarpa* (version 3.0) genome was used as a reference for clean reads mapping using TopHat (version: 2.0.9) (Trapnell *et al*., 2009). Mismatches of three bases or less and multihits of no more than one base were allowed in the alignment. We used three filter processes to identify the osmotic stress‐responsive lncRNA candidates. First, the length of transcriptional units (TUs) had to be longer than 200 bp. Second, the longest open reading frame (ORF) of the TU had to be smaller than 300 bp (the longest ORF was predicted by OrfPredictor; http://proteomics.ysu.edu/tools/OrfPredictor.html) (Min *et al*., 2005). Sense and antisense strands of the TUs were used for prediction. The Coding Potential Calculator (CPC) and Coding–Non‐Coding Index (CNCI) were used to assess the protein‐coding potential of a transcript based on two criteria: a CPC score < 0 and a CNCI < 0 (Kong *et al*., 2007; Sun *et al*., 2013a,b). Finally, the lncRNA candidate sequences were mapped to the genome and full‐length lncRNAs located in promoter regions were annotated as PROMPTs.

### Sequence and structural motif search

Conserved sequence motif searches in a group of lncRNAs were carried out by MEME (http://meme-suite.org/tools/meme) (Bailey *et al*., 2009). For the lncRNAs selected by the target's GO term, we predicted the conserved structural motifs in grouped lncRNAs using *RNApromo* (https://genie.weizmann.ac.il/pubs/rnamotifs08/rnamotifs08_predict.html) (Rabani *et al*., 2008). The details of sequence and structural motif analysis are presented in the supplemental data.

### Quantitative PCR analysis

To validate the expression patterns of PROMPTs acquired by high‐throughput sequencing, we performed qPCR for 40 osmotic stress‐responsive PROMPTs with different expression patterns (Table S10). We found a significant correlation between transcript abundance measured by qPCR and RNA‐seq (*r *=* *0.71, *P *<* *0.001), indicating the reliability of the RNA‐seq data (Figure S10). All primers used for candidate genes and PROMPTs are listed in Table S11. qPCR was performed using an ABI Step One Plus instrument, and the results were subjected to the following calculations: sample cycle threshold (Ct) values were determined and standardized relative to the endogenous control gene (*ACTIN*), and the 2^−ΔΔCT^ method was used to calculate the relative changes in gene expression based on the qPCR data (Livak and Schmittgen, 2001). A melting curve was used to check the specificity of each amplified fragment. For all reactions, triplicate technical and biological repetitions were performed for each individual. After amplification, the PCR products were sequenced to check the specificity of the primer sets.

### Differential expression and co‐expression analyses

Cuffdiff was used to calculate fragments per kilobase of exon per million fragments mapped (FPKMs). The FPKMs of lncRNAs and genes were computed by summing the FPKMs of transcripts in each transcript group. Cuffdiff provides statistical routines for determining differential expression in transcript data using a model based on the negative binomial distribution (Trapnell *et al*., 2010). Differential expression analysis of two conditions or groups was performed using the DESeq R package (1.8.3) (Heidelberg, Germany). The *P*‐values were adjusted using the Benjamini & Hochberg method. Differences of mRNA, lncRNA and miRNA levels were considered statistically significant at a fold change >2 or <0.5 and *P *<* *0.01.

Gene co‐expression network analysis has been increasingly used to identify the biological functions of lncRNAs and their potential subnetworks for *trans* targets (Liao *et al*., 2011). One important end product of co‐expression networks is the construction of gene modules composed of highly interconnected genes. To identify gene co‐expression modules from 15 the RNA sequencing data (accession numbers SRR5127346, SRP095225, SRP073689 and SRP060593), the WGCNA package for R was used to calculate the correlation coefficient (Langfelder and Horvath, 2008). Normalized lncRNAs and mRNA expression values were used for co‐expression analysis. One‐step network construction with unsigned correlations type and consensus module detection was used for co‐expression network construction. All other WGCNA parameters remained at their default settings. Assessment of module quality was assisted by examining trend plots of *Z*‐score normalized expression values for all genes in a given module. The mRNA co‐expression modules were used for GO enrichment analysis of the lncRNAs. Statistical significance for enrichment of genes was assessed using the hypergeometric distribution.

### Treatment with LDH–lncRNA conjugates

Bulk Mg‐Al‐lactate LDH was synthesized using a coprecipitation method and delaminated in water into nano‐scale sheets. The delaminated LDH‐lactate is denoted as LDH‐lactate‐NS with a final concentration of 1 mg/mL. Candidate lncRNAs were artificially synthesized (Table S8). These lncRNAs were dissolved in distilled water to a concentration of 1 mg/mL (Bao *et al*., 2016). The LDH‐lactate‐NS colloid in MS was added dropwise to the lncRNAs at a ratio of 3 : 1 (v:v) followed by gentle mixing. RNase inhibitor was added to a final concentration of 0.4 U/μL. The mixture was incubated for 1 h to form the LDH‐lactate‐NS–lncRNA conjugate. Then, 100 μL of LDH‐lactate‐NS–lncRNA conjugate was added to liquid MS medium for transport of the RNA into plant roots (Figure S6). Poplar seedlings were cultured in MS medium. For LDH–lncRNA conjugates treatment, poplar seedling lateral roots were dipped in liquid MS medium containing 0.2 μg/μL of the LDH‐lactate‐NS–lncRNA conjugate. After incubation at room temperature for 3 h, the roots were rinsed several times with a standard growth medium and then stored at −80 °C for expression analysis. For the osmotic treatment with 30% PEG6000, the poplar seedling lateral roots with LDH‐lactate‐NS–lncRNA conjugate were dipped in liquid MS medium containing with 30% PEG6000 (Figure S11). After incubation at room temperature for 1 h, the roots were stored at −80 °C for expression analysis.

## Conflict of interest

The authors declare no conflict of interest.

## Supporting information


**Figure S1** *Cis*‐regulatory functions of the osmotic stress‐responsive PROMPTs.
**Figure S2** Transcript abundance of PROMPTs and downstream protein‐coding genes under osmotic stress.
**Figure S3** Co‐expression network of PROMPTs and mRNA.
**Figure S4** Secondary structure of *PROMPT_1281‐Hap1* and *PROMPT_1281‐Hap2*.
**Figure S5** Nucleotide substitution rates are suppressed within PROMPT transcripts.
**Figure S6** Schematic diagram of lncRNA interference and lncRNA enhance.
**Figure S7** Secondary structure of *PROMPT_1281‐Hap1* and *PROMPT_1281‐Hap2* with mutated loop 4.
**Figure S8** The secondary structure and spatial effect of PROMPTs regulate transcript of targets.
**Figure S9** Correlation of qPCR and RNA‐seq data.
**Figure S10** Microscopic images of intact poplar root cells under FITC‐PROMPT‐LDH treatment.
**Figure S11** Microscopic images of intact poplar root cells under the FITC‐PROMPT‐LDH treatment.

**Table S1** Mapping results statistics.
**Table S2** Information on the osmotic stress‐responsive lncRNAs in poplar.
**Table S3** Information on the PROMPTs and downstream genes in poplar.
**Table S4** Differentially expressed lncRNAs of poplar under osmotic stress.
**Table S5** Differentially expressed PROMPTs of poplar under osmotic stress.
**Table S6** Annotation of the motifs of osmotic stress‐responsive PROMPTs.
**Table S7** Co‐expression of osmotic stress‐responsive PROMPTs and genes.
**Table S8** Sequences of PROMPTs used for lncRNAe and lncRNAi analysis.
**Table S9** Go term enrichment of NM1.
**Table S10** qPCR primer sequences.
**Table S11** Primer list of *PROMPT_1281* and their targets.
