# Supplementary material for: Osmotic stress‐responsive promoter upstream transcripts (PROMPTs) act as carriers of MYB transcription factors to induce the expression of target genes in Populus simonii
Source: Plant Biotechnol J. 2018 Jun 28;17(1):164–77. doi: 10.1111/pbi.12955 (PMC6330638; doi:10.1111/pbi.12955)
Supplement: Supplementary file 1 — Figure S1 Cis‐regulatory functions of the osmotic stress‐responsive PROMPTs. Figure S2 Transcript abundance of PROMPTs and downstream protein‐coding genes under osmotic stress. Figure S3 Co‐expression network of PROMPTs and mRNA. Figure S4 Secondary structure of PROMPT_1281‐Hap1 and PROMPT_1281‐Hap2. Figure S5 Nucleotide substitution rates are suppressed within PROMPT transcripts. Figure S6 Schematic diagram of lncRNA interference and lncRNA enhance. Figure S7 Secondary structure of PROMPT_1281‐Hap1 and PROMPT_1281‐Hap2 with mutated loop 4. Figure S8 The secondary structure and spatial effect of PROMPTs regulate transcript of targets. Figure S9 Correlation of qPCR and RNA‐seq data. Figure S10 Microscopic images of intact poplar root cells under FITC‐PROMPT‐LDH treatment. Figure S11 Microscopic images of intact poplar root cells under the FITC‐PROMPT‐LDH treatment. [file PBI-17-164-s001.doc]

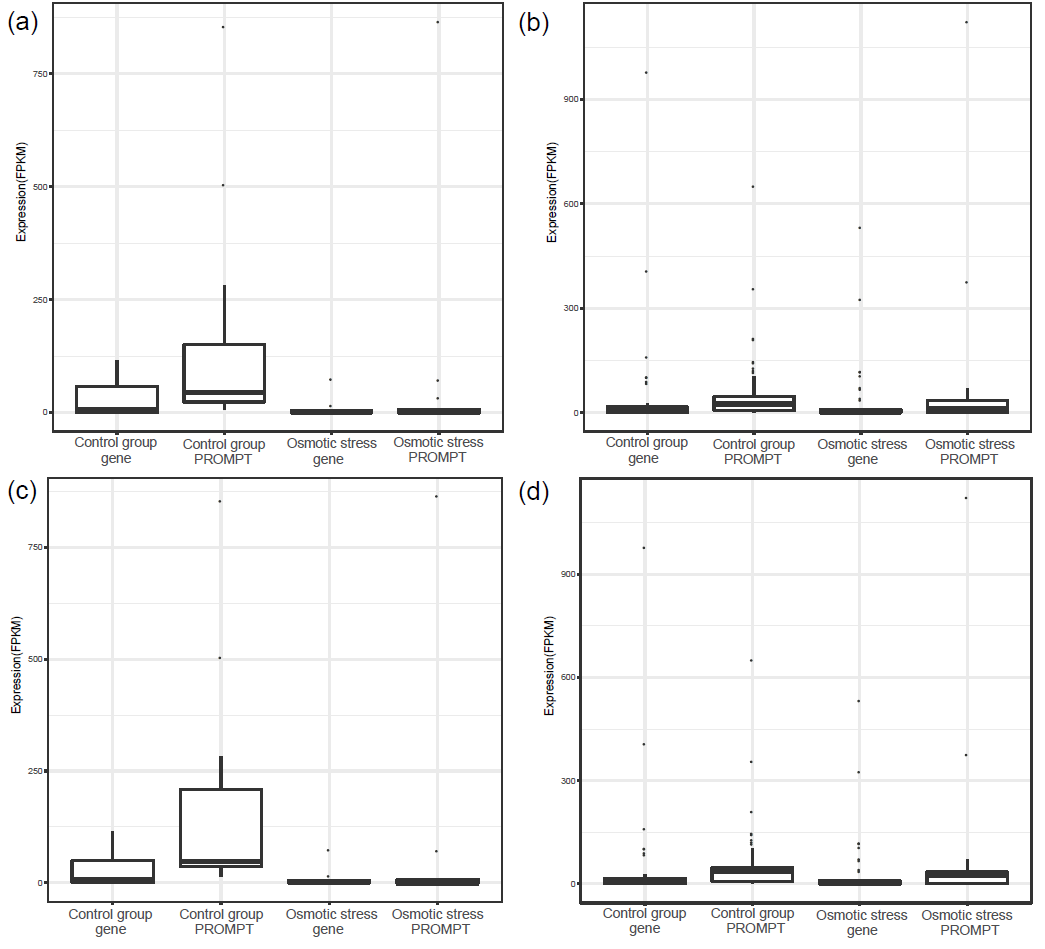


**Figure S1. *Cis*-regulatory functions of the osmotic-responsive PROMPTs**. (a) The expression of PROMPTs and genes transcribed from sense strands. (b) The expression of PROMPTs and genes transcribed from antisense strands. (c) Transcript abundance of sense/sense pairs of PROMPTs and genes. (d) Transcript abundance of antisense/antisense pairs of PROMPTs and genes.


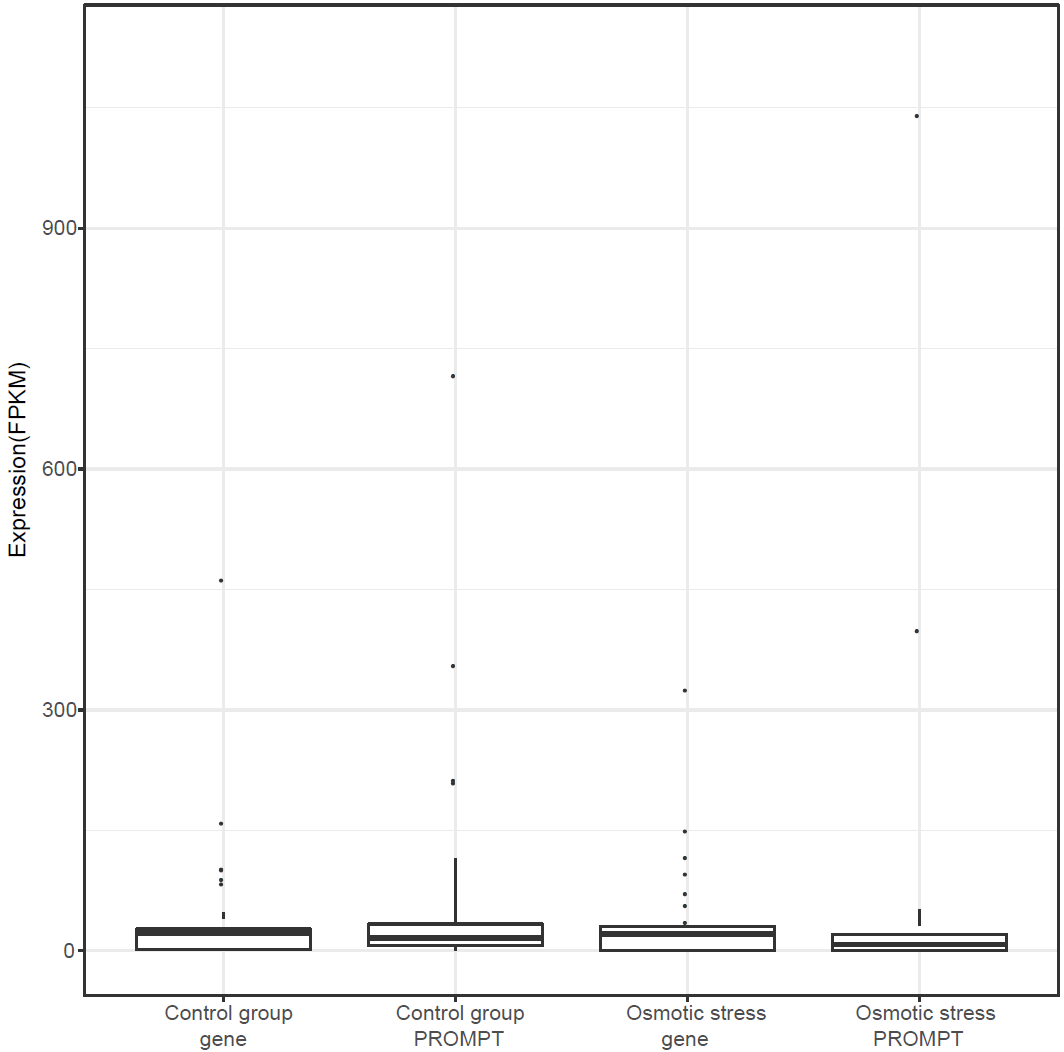


**Figure S2. Transcript abundance of PROMPTs and downstream protein-coding genes under osmotic stress.
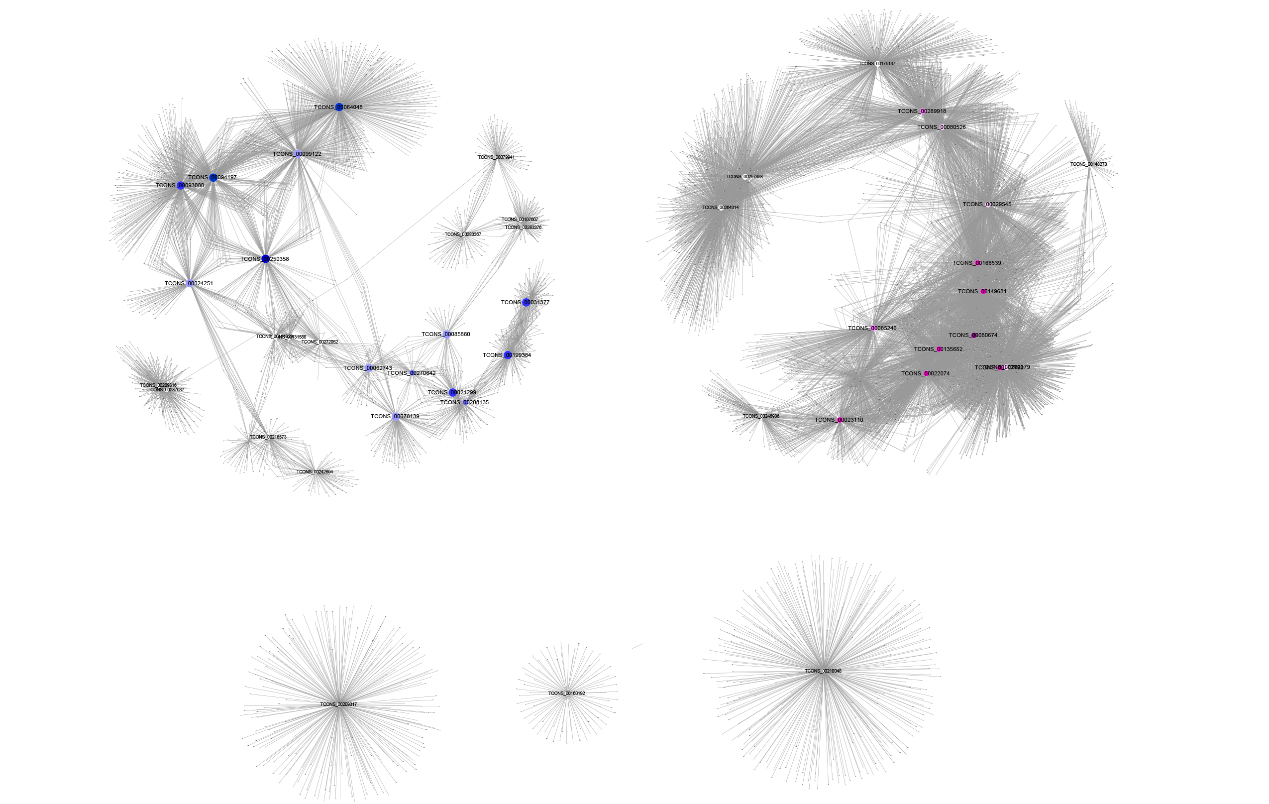
**

**Figure S3. Co-expression network of PROMPTs and mRNA.**


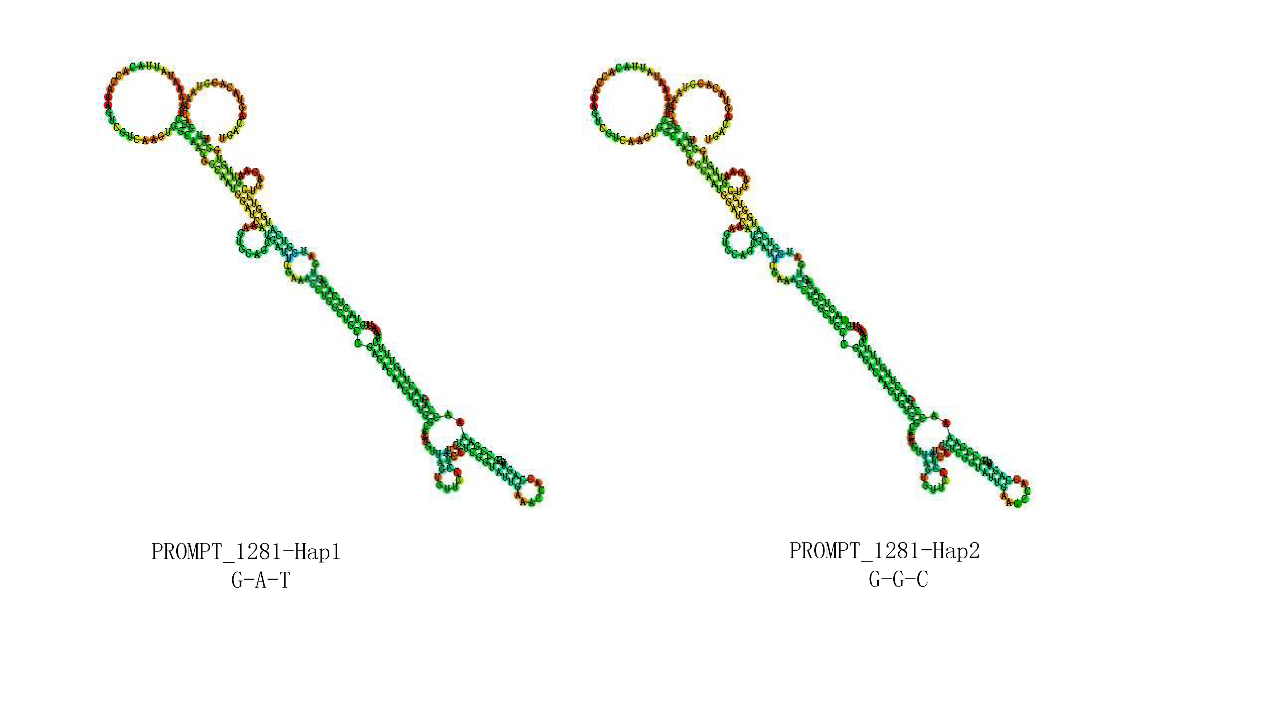


**Figure S4. Secondary structure of PROMPT_1281-Hap1 and PROMPT_1281-Hap2.**


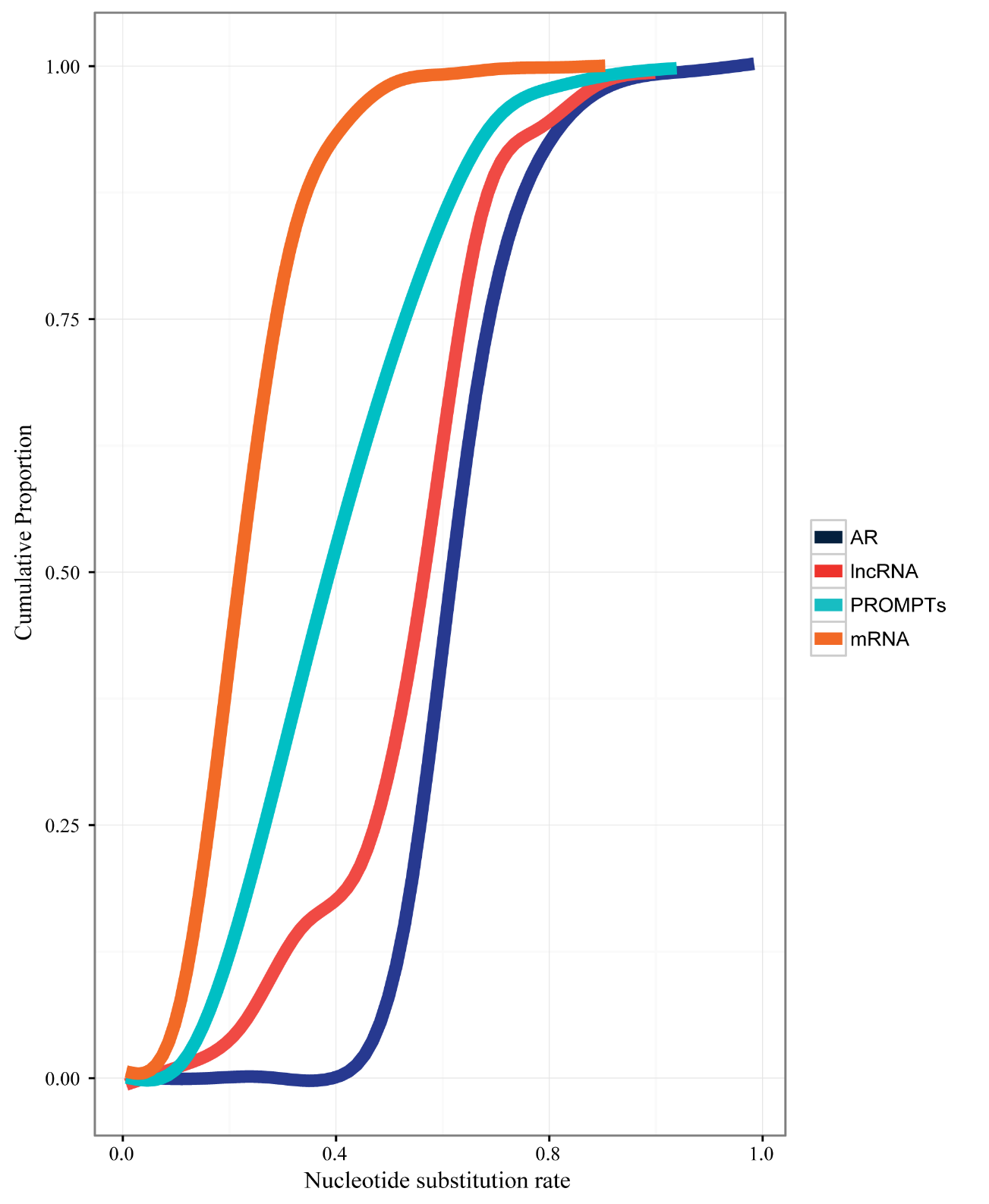


**Figure S5**. **Nucleotide substitution rates are suppressed within PROMPT transcripts.** Panel shows the cumulative distribution of substitutions measured on lncRNA transcripts (red curve), mRNA transcripts (orange curve), PROMPT transcripts (blue/green curve), and the same rates measured on nearby neighboring ancestral repeat sequences of matched length (blue curve).

**Figure S6. Schematic diagram of lncRNA interference and lncRNA enhancement.** FITC represent fluorescein isothiocyanate. LDH-lactate-NS represent delaminated lactate containing layered double hydroxide nanosheets.


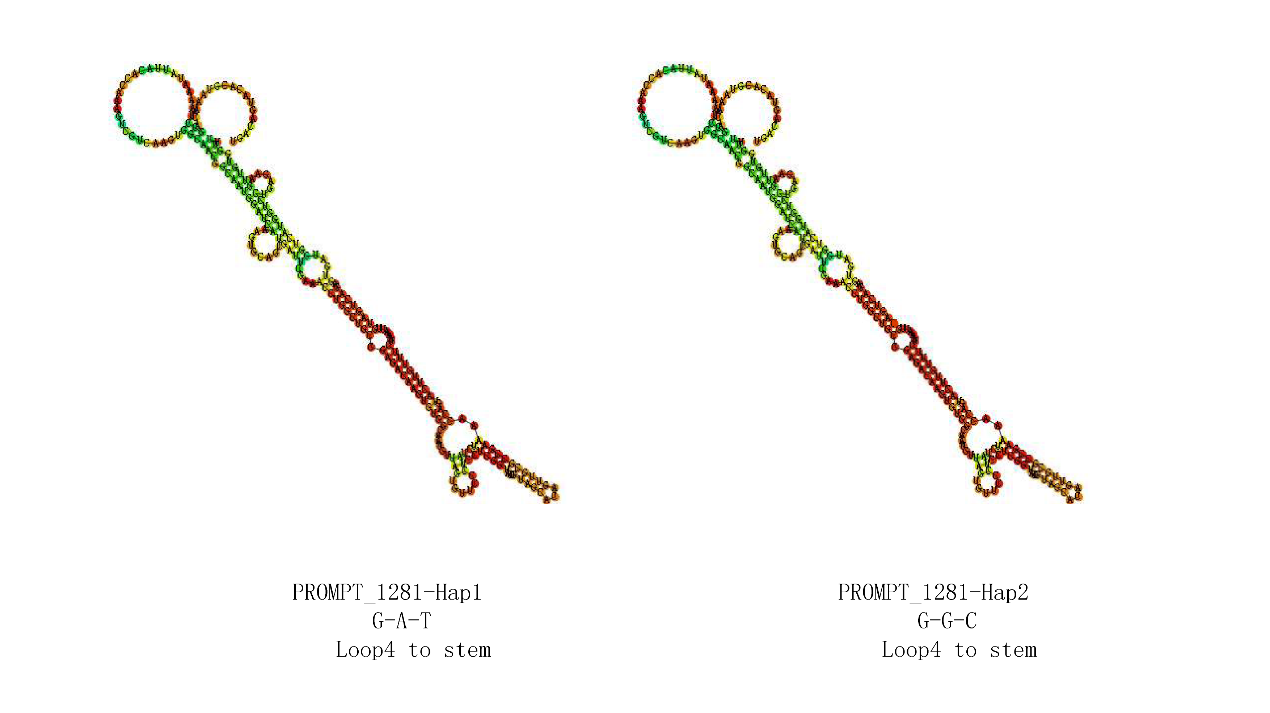


**Figure S7. Secondary structure of PROMPT_1281-Hap1 and PROMPT_1281-Hap2 with mutant Loop 4.**
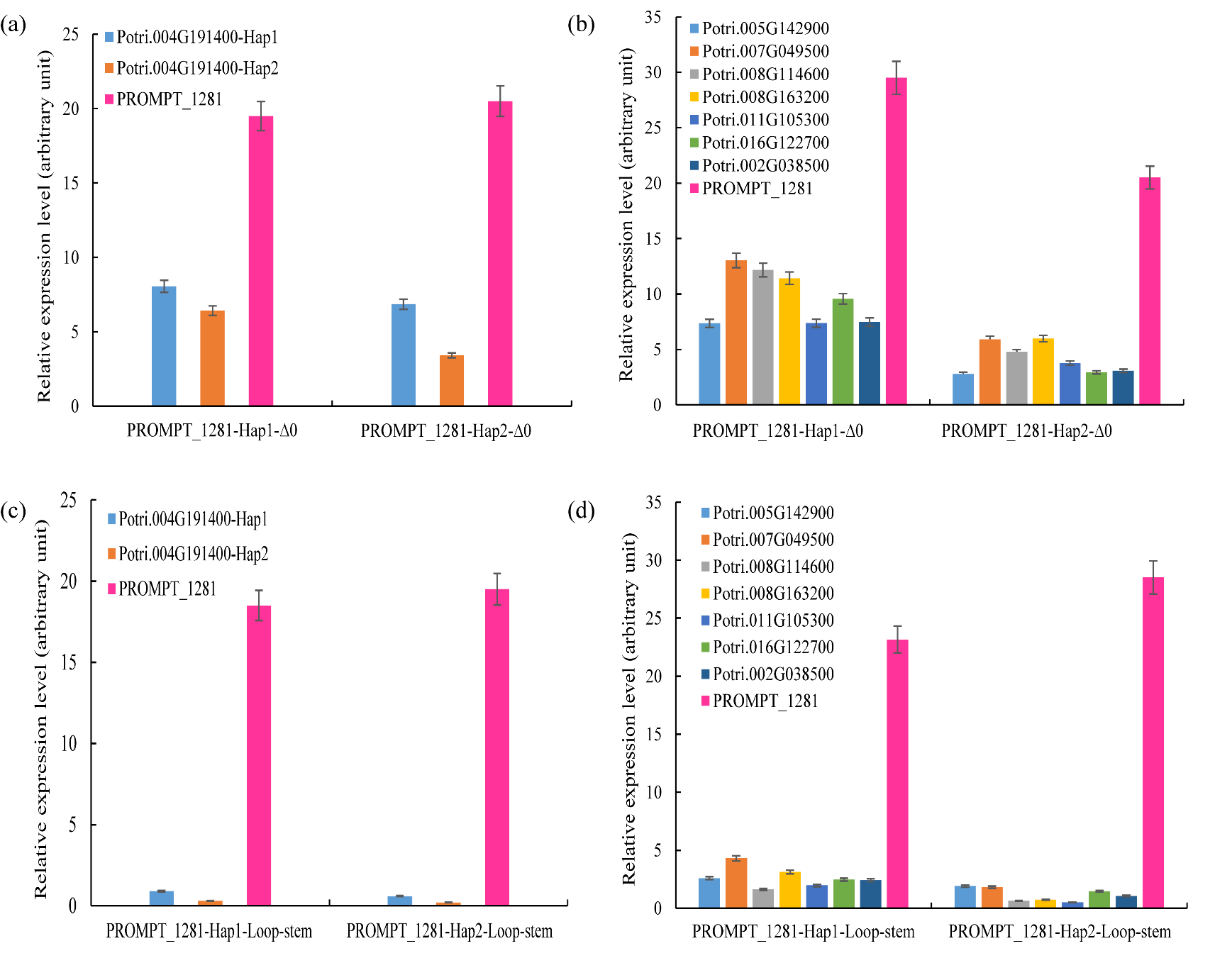


**Figure S8.** **The secondary structure and** **spatial effect of PROMPTs regulate transcript of targets**. (a) Expression pattern of *cis* targets with enhanced abundance of *PROMPT_1281* lacking loop 0. (b) Expression pattern of *trans* targets with enhanced abundance of *PROMPT_1281* lacking loop 0. ∆0 represents the sequences of loop 0 that were deleted in *PROMPT_1281.* (c) Expression pattern of *cis* targets with enhanced abundance of *PROMPT_1281* with mutant loop 4. (d) Expression pattern of *trans* targets with enhanced abundance of *PROMPT_1281* with mutant loop 4.

**Figure S9. Relative leaf water content of poplar in response to osmotic stress.**

Error bars represent standard error. Asterisk indicate significant differences at P < 0.01.


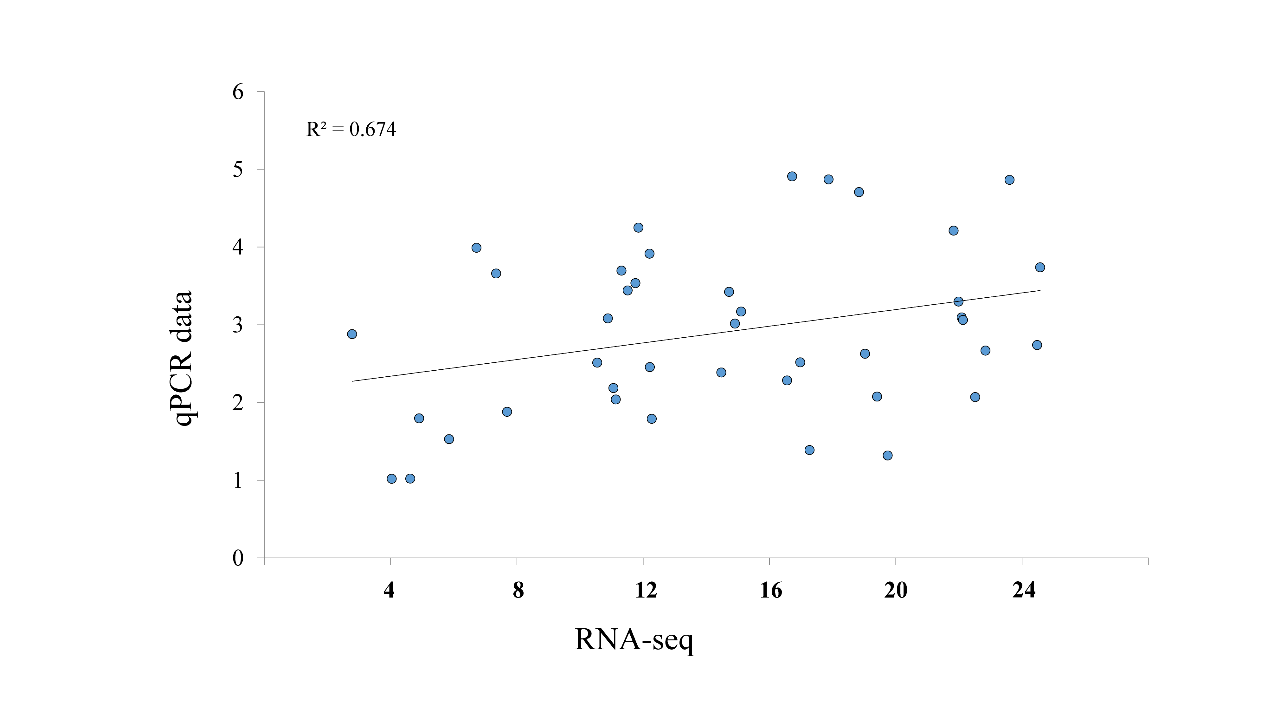


**Figure S10. Correlation of qPCR and RNA-seq data.**

**Figure S11. Microscopic images of intact poplar root cells under the FITC-PROMPT-LDH treatment**. FITC-PROMPT represents the fluorescence microscopic images of intact poplar root cells 3 h post treatment with FITC-lncRNAs after washing. Scale bars = 60 μm. FITC-PROMPT-LDH represents the fluorescence microscopic images of intact poplar root cells 3 h post treatment with FITC-PROMPT-LDH after washing. Green light represents the fluorescence of LDH-lactate-NS-PROMPT-FITC from the cytosol of poplar root cells, meaning that LDH-lactate-NS delivered synthetic lncRNAs into the intact roots. Scale bar = 100μm.
